# Application of an Adaptive, Digital, Game-Based Approach for Cognitive Assessment in Multiple Sclerosis: Observational Study

**DOI:** 10.2196/24356

**Published:** 2021-01-20

**Authors:** Wan-Yu Hsu, William Rowles, Joaquin A Anguera, Chao Zhao, Annika Anderson, Amber Alexander, Simone Sacco, Roland Henry, Adam Gazzaley, Riley Bove

**Affiliations:** 1 Department of Neurology Weill Institute for Neurosciences University of California, San Francisco San Francisco, CA United States; 2 Neuroscape University of California, San Francisco San Francisco, CA United States; 3 Department of Psychiatry University of California, San Francisco San Francisco, CA United States; 4 Department of Physiology University of California, San Francisco San Francisco, CA United States

**Keywords:** cognition, digital health, mHealth, multiple sclerosis, cognitive assessment, video game

## Abstract

**Background:**

Cognitive impairment is one of the most debilitating manifestations of multiple sclerosis. Currently, the assessment of cognition relies on a time-consuming and extensive neuropsychological examination, which is only available in some centers.

**Objective:**

To enable simpler, more accessible cognitive screening, we sought to determine the feasibility and potential assessment sensitivity of an unsupervised, adaptive, video game–based digital therapeutic to assess cognition in multiple sclerosis.

**Methods:**

A total of 100 people with multiple sclerosis (33 with cognitive impairment and 67 without cognitive impairment) and 24 adults without multiple sclerosis were tested with the tablet game (EVO Monitor) and standard measures, including the Brief International Cognitive Assessment for Multiple Sclerosis (which included the Symbol Digit Modalities Test [SDMT]) and Multiple Sclerosis Functional Composite 4 (which included the Timed 25-Foot Walk test). Patients with multiple sclerosis also underwent neurological evaluations and contributed recent structural magnetic resonance imaging scans. Group differences in EVO Monitor performance and the association between EVO Monitor performance and standard measures were investigated.

**Results:**

Participants with multiple sclerosis and cognitive impairment showed worse performance in EVO Monitor compared with participants without multiple sclerosis (*P*=.01) and participants with multiple sclerosis without cognitive impairment (all *P*<.002). Regression analyses indicated that participants with a lower SDMT score showed lower performance in EVO Monitor (*r*=0.52, *P*<.001). Further exploratory analyses revealed associations between performance in EVO Monitor and walking speed (*r*=–0.45, *P*<.001) as well as brain volumetric data (left thalamic volume: *r*=0.47, *P*<.001; right thalamic volume: *r*=0.39, *P*=.002; left rostral middle frontal volume: *r*=0.28, *P*=.03; right rostral middle frontal volume: *r*=0.27, *P*=.03).

**Conclusions:**

These findings suggest that EVO Monitor, an unsupervised, video game–based digital program integrated with adaptive mechanics, is a clinically valuable approach to measuring cognitive performance in patients with multiple sclerosis.

**Trial Registration:**

ClinicalTrials.gov NCT03569618; https://clinicaltrials.gov/ct2/show/NCT03569618

## Introduction

Cognitive impairment (CI) occurs in 30% to 70% of people with multiple sclerosis (MS) and has a profound influence on a patient’s personal functioning, social interaction, employment, and overall quality of life [1,2]. Being able to effectively detect CI is essential to better managing further decline [3] and helping patients navigate problems related to their daily living. The most commonly affected cognitive domains are processing speed, attention, executive function, and memory [4,5]. Currently, clinical cognitive assessment relies on a comprehensive neuropsychological examination, which is time-consuming and extensive. The examination results may be affected by patient fatigue or loss of engagement. Given the large interindividual variability in the pattern of CI in MS [6,7], the traditional nonadaptive assessments may overlook cognitive deficiencies [8]. Taking advantage of technology, digital tools offer a platform to integrate personalizing features, including adaptive staircase algorithms to video game–style mechanics for cognitive assessment [8-15]. This approach can mitigate potential ceiling and floor effects when interindividual variability is high, and leads to more reliable assessments that can be completed in a timely manner [8,16]. Furthermore, digital tools can be easily applied in different settings, including patients’ homes, which substantially improves health care accessibility for patients who have difficulties with travel to the clinic due to cognitive or physical disabilities [14,17,18].

Digital tools have been used for functional assessment, rehabilitation, and health care monitoring in clinical populations such as stroke [19,20], schizophrenia [21], depression [10,12], attention-deficit/hyperactivity disorder [15,22-24], and neurodevelopmental disorders [8,9]. In MS, studies have demonstrated that by using digital tools, health care services can be delivered effectively [25,26] and even that comprehensive neurological exams can be performed remotely [27]. However, to the best of our knowledge, applications of serious video game–based digital tools incorporating closed-loop adaptation mechanics as cognitive assessments for people with MS have not been assessed.

We previously evaluated whether a video game–based digital tool, EVO-AKL-T01 (Akili), could improve cognition in MS [17,18]. After these studies, we looked to better characterize cognitive function using similar tools based on these cognitive therapeutics. EVO Monitor (Akili) was developed based on findings that a precursor (NeuroRacer), embedded with adaptive algorithms, is sensitive to age-related cognitive decline across the lifespan and can enhance cognitive control [28]. EVO Monitor, which includes some of NeuroRacer’s early closed-loop features, is a novel, tablet-based, digital platform that is incorporated with video game mechanisms, visual and auditory feedback, adaptive algorithms, and sophisticated graphics that is designed to assess executive function, attention, and information processing speed for clinical populations. Specifically, it consists of 3 tasks: perceptual discrimination, visuomotor tracking, and multitasking. In the perceptual discrimination task, the participants complete a go/no go–like paradigm, in which they tap the iPad screen for correctly colored target stimuli while ignoring distracting targets. The visuomotor tracking task requires the participant to tilt the iPad to steer an avatar around obstacles. The multitasking task requires participants to perform both perceptual discrimination and visuomotor tracking concurrently. The tool is designed to challenge attention, goal management, and information processing speed in the setting of interference. EVO Monitor has been validated as a tool to assess cognitive ability in children with and without neurodevelopmental disabilities. For example, it can differentiate between the performance of children with or without 16p11.2 BP4-BP5 deletion and healthy controls, whereas traditional nonadaptive cognitive assessments overlook group differences [8].

In this study, we aimed to determine the feasibility and potential assessment sensitivity of EVO Monitor as an unsupervised, tablet game–based approach with adaptive algorithms to assess cognition in MS. In this context, 100 people with MS and 24 adults without MS were tested with EVO Monitor and standard measures, including the written version of Symbol Digit Modalities Test (SDMT), the most sensitive test for detecting cognitive involvement in the MS course [29,30]. Group-level differences were examined to evaluate whether EVO Monitor is sensitive to differences among participants with MS with and without CI as well as participants without MS. The association between performance in EVO Monitor and standard measures was investigated. Furthermore, since cognition in MS has been shown to correlate with physical fitness, where aerobic capacity and muscular strength outcomes are associated with cognitive processing speed and inhibitory control [31,32], and magnetic resonance imaging (MRI) measures have shown that thalamus damage is associated with the presence of CI, and frontal lesion is associated with executive function [33,34], exploratory analyses of the correlation between performance in EVO Monitor and both physical measures and MRI volumetric data (thalamus and frontal lobes) were conducted to understand whether cognitive performance assessed by a video game–based digital tool (EVO Monitor) would be associated with physical activity and structural MRI measures.

## Methods

### Participants

A total of 100 adults with a diagnosis of clinically isolated syndrome or MS [35] (mean age: 52.2, standard error of the mean [SEM] 1.24 years) were recruited from the University of California, San Francisco (UCSF) Multiple Sclerosis and Neuroinflammation Center. Patients with clinical relapses or steroid use in the past month or with severe visual, cognitive, or motor impairment that would preclude the use of a tablet-based tool were excluded. A group of 24 adults without MS (non-MS) (mean age: 46.0, SEM 3.72 years) with no chronic autoimmune diseases were also recruited from the UCSF staff, willing family members of patients in the clinic, and other eligible and willing volunteers. All participants with MS were recruited as part of studies to determine the feasibility [18] and preliminary efficacy [17] of the EVO platform as a digital therapeutic to improve processing speed in people with MS. The analysis of this study was based on baseline performance data (ie, before any cognitive intervention) of our feasibility [18] and efficacy [17] trials. All procedures performed in the study involving human participants were approved by the Committee for Human Research at the University of California, San Francisco. Written informed consent was obtained from each participant. The trial is registered with ClinicalTrials.gov (NCT03569618).

### Task Description

#### Standard Measures

The Brief International Cognitive Assessment for Multiple Sclerosis is a cognitive assessment tool that is validated in MS populations as compared with participants without MS [36]. It is a standardized, internationally validated battery [37] including (1) the SDMT, a widely used measure of attention and information processing speed in MS [29,30]; (2) the California Verbal Learning Test Second Edition, a verbal memory immediate recall test [38]; and (3) the Brief Visuospatial Memory Test Revised, a visual memory immediate recall test [39]. Serial versions of all tests were used to minimize practice effects [40].

The MS Functional Composite 4 evaluates 4 key MS-related functional domains [41]: walking speed (Timed 25-Foot Walk [T25FW]), upper extremity function (Nine-Hole Peg Test), vision (Sloan low-contrast letter acuity test), and cognition (with a test of information processing speed, attention, and working memory [Paced Auditory Serial Addition Task] [42]).

#### EVO Monitor

**Figure 1:**
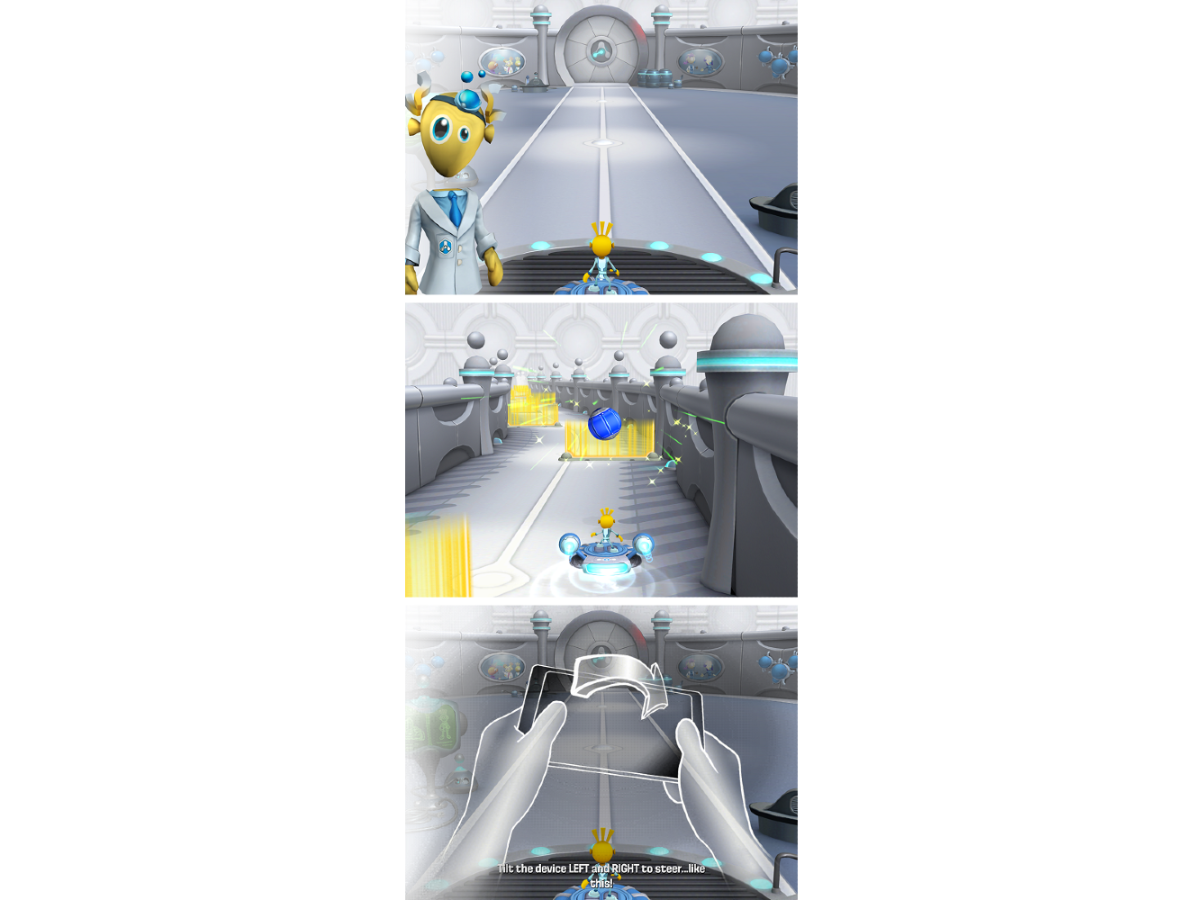
Screenshot of the EVO Monitor cognitive assessment program. The participants are instructed to respond to colored target stimuli by tapping the iPad screen while navigating a character along a dynamically moving road and avoiding walls and obstacles by tilting the iPad. Copyright © 2020-2021, Akili Interactive Labs, Inc. All rights reserved.

EVO Monitor is a digital cognitive assessment developed by Akili Interactive Labs (Akili, Boston) to assess cognitive function, including attention and related cognitive control processes in clinical populations (Figure 1). The program is an immersive action video game that has been engineered with adaptive algorithms to target fronto-parietal brain networks fundamentally linked to attentional control among other aspects of cognition. It was developed based on the principles of NeuroRacer, an innovative cognitive intervention that is sensitive to age-related cognitive decline [28]. EVO Monitor comprises 3 tasks: perceptual discrimination, visuomotor tracking, and multitasking. In the perceptual discrimination task, the participants are instructed to respond to colored target stimuli by tapping the iPad screen while ignoring distractors. In the visuomotor tracking task, the participants navigate a character along a dynamically moving road while avoiding walls and obstacles by tilting the iPad. The multitasking task requires participants to perform both perceptual discrimination and visuomotor tracking at the same time until participants complete a minimum number of trials and reach a stable level of performance.

The adaptive algorithms change game difficulty on a trial-by-trial basis for both the perceptual discrimination (adapting the response window for a target) and visuomotor tracking (adapting the speed of the forward path), with real-time feedback making the participants aware of their performance. More specifically, the closed-loop adaptive algorithm makes proportional changes in gameplay difficulty to keep the player’s performance at an approximate 80% rate of accuracy based on adaptive psychometric principles [43-45], which ensures that task difficulty is equated across participants [45,46] and enhances participant engagement [47]. It takes approximately 7 minutes to complete the tasks, with a specified number of both correct and incorrect trials allowing the adaptive algorithm to settle on a prescribed level of difficulty (ie, threshold level) that would converge on a consistent accuracy rate. The threshold level represents task performance, as it indicates the task difficulty at which the participants achieve approximately an 80% rate of accuracy. 

At the beginning of the EVO Monitor session, a brief instruction is given on the iPad screen with a short practice to ensure the participant understands the tasks, followed by one session of the actual assessment, which takes about 7 minutes, with a task order of multitasking, perceptual discrimination, visuomotor tracking, and multitasking. Each task is about 1.5 to 2 minutes with no break in between. Although the assessment is self-guided, a study coordinator sat in with participants to ensure they were following the instructions. None of the participants had played EVO Monitor before participation. Since EVO Monitor continuously monitors the user’s performance at a rate of 30 frames per second, the measured reaction time, perceptual discrimination task sensitivity (eg, hit and false alarm rates), and visuomotor tracking performance generate 39 basic performance metrics. Among the 39 metrics extracted from participants’ navigation of EVO Monitor, we prespecified the calculated threshold levels during both single (perceptual discrimination, visuomotor tracking) and multitasking conditions as attentional measures, according to previous studies [8,16].

#### Basic Reaction Time

To ensure that any observed differences in cognitive measures between groups were not due to differences in motoric speed, we assessed basic response speed of participants on a simple task with minimal loading on executive function skills [28]. This task was designed to index the motoric speed, and the measured data were only included in the analyses as a covariate to control for potential motor speed deficits in participants with MS. In this task, participants were instructed to respond to a target stimulus (40 trials) as fast as they could by tapping a button on an iPad platform. Similar to EVO Monitor, this task used adaptive algorithms that modulate the challenge level of the task on a trial-by-trial basis based on individual performance. Only data from the dominant hand were included as each participant’s basic reaction time (BRT) in the following analyses.

#### Clinically Acquired MRI Measures

Clinically or research-acquired brain isotropic T1 and T2 fluid-attenuated inversion recovery images were available for 56 participants with MS (16/56, 29% with 1.5-T and 40/56, 71% with 3-T MRIs) at a mean of 76.0 (SEM 33.9) days before the study visit. Lesion segmentation was performed using the LST (lesion segmentation toolbox) lesion probability algorithm 2.0 DICOM (Digital Imaging and Communications in Medicine) v1.4 segmentation pipeline, which creates lesion probability maps, masks, and labels. These were then manually validated by an expert radiologist (SS). Volumetric analysis was performed from T1 anatomical images using 3 complementary tools: FreeSurfer 5.3 and ANTs Morphology 2.1.0 [48], used to segment tissue into cerebrospinal fluid, cortical grey matter, subcortical grey matter, white matter, brainstem, and cerebellum, and Mindboggle 1.0 [49], which combines the morphology outputs of FreeSurfer and ANTs to generate volume images and tabular information for further analysis. Bilateral thalamic and frontal lobe volumetric measures were normalized to individual intracranial volume [50] for exploratory analyses of association between performance in EVO Monitor and MRI volumetric measures. The selection of the thalamus and frontal lobe as regions of interest was based on ample evidence indicating an association between atrophy in these regions and cognitive dysfunction in MS [33,34].

### Statistical Analysis

In order to evaluate whether EVO Monitor is sensitive to differences among participants with MS with and without CI as well as participants without MS, participants with MS were divided into 2 subgroups (ie, CI and non-CI) according to their baseline SDMT *z* scores. We characterized the participants with a SDMT *z* score lower than –1 based on published normative data [51] as having CI. Group-level differences were assessed with one-way analysis of covariance (ANCOVA) with age, sex, years of education, and BRT as covariates to control for potential differences in demographic features and motoric quickness. Two-tailed Student *t* tests were carried out for post hoc comparisons when appropriate. To discern the association between EVO Monitor and standard measures, Pearson correlation analyses were performed between performance in EVO Monitor and SDMT. In an exploratory analysis, the correlation between performance in EVO Monitor and both physical MS measures (T25FW) and MRI volumetric data (thalamus and frontal lobes) was assessed with Pearson correlation analyses. Partial correlation analyses including age, sex, years of education, and BRT as covariates were applied when appropriate. All numerical data are presented as the mean and SEM. The statistical analyses were performed using IBM SPSS Statistics version 22.0 (IBM Corp). The significance of the statistical level was set at *P*≤.05.

## Results

### Participant Characteristics

**Figure 2:**
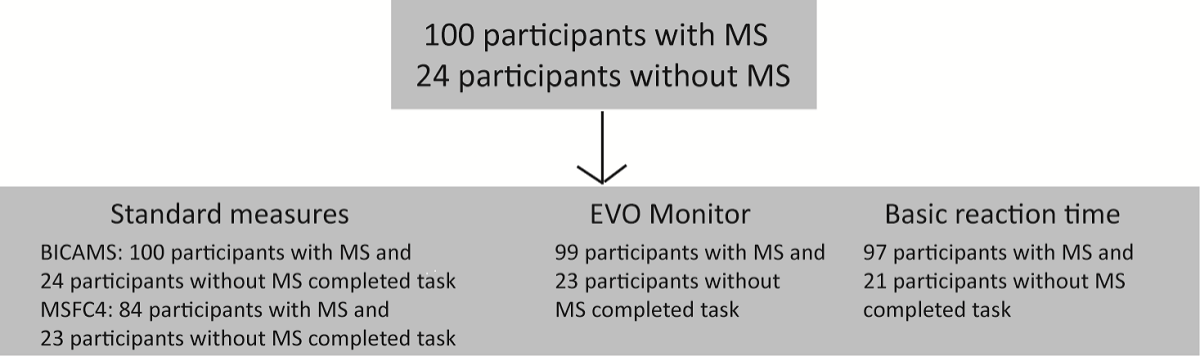
Study task completion rate. BICAMS: Brief International Cognitive Assessment for Multiple Sclerosis; MS: multiple sclerosis; MSFC: Multiple Sclerosis Functional Composite.

A total of 100 participants with MS and 24 participants without MS were enrolled in the study. For analysis purposes, the 100 participants with MS were divided into CI (n=33) and non-CI (n=67) subgroups. Table 1 summarizes their clinical and demographic characteristics. Figure 2 details the completion rate for each test.

**Table 1:** Demographic and clinical characteristics of participants.

Characteristic		MS^a^		Non-MS (n=24)
		CI^b^ (n=33)	Non-CI (n=67)	
Age (years), mean (SEM)		48.96 (2.29)	53.80 (1.44)^c^	46.04 (3.72)
Sex (female), n (%)		24 (73)	51 (76)	12 (50)
Education (years), mean (SEM)		16.36 (0.41)	16.70 (0.30)	16.16 (0.41)
Handedness (right-handed), n (%)		30 (90)	59 (88)	24 (100)
**Race, n (%)**				
	White	29 (88)	56 (84)	19 (79)
	Black or African American	1 (3)	4 (6)	1 (4)
	Other or unknown	3 (9)	7 (10)	4 (17)
SDMT^d^ score, mean (SEM)		35.18 (6.43)	49.79 (0.98)^e^	51.20 (2.65)^f^
SDMT *z* score, mean (SEM)		–1.53 (0.06)	0.14 (0.09)^e^	0.26 (0.20)^f^
EDSS^g^, median (IQR)		4 (2.75)	3 (2)	N/A^h^
Disease duration (years), mean (SEM)		11.65 (1.54)	13.27 (1.05)	N/A
**MS subtype, n (%)**				
	Relapsing-remitting	26 (79)	48 (72)	N/A
	Primary progressive	2 (6)	9 (13.5)	N/A
	Secondary progressive	4 (12)	7 (10.5)	N/A
	CIS^i^	0 (0)	2 (3)	N/A
	Unknown	1 (3)	1 (1)	N/A

^a^MS: multiple sclerosis.

^b^CI: cognitive impairment.

^c^*P*=.04 for the comparison between non-MS and non-CI groups.

^d^SDMT: Symbol Digit Modalities Test.

^e^*P*<.001 for the comparison between CI and non-CI groups.

^f^*P*<.001 for the comparison between CI and non-MS groups.

^g^EDSS: Expanded Disability Status Scale.

^h^N/A: not applicable.

^i^CIS: clinically isolated syndrome.

### Group Differences

**Figure 3:**
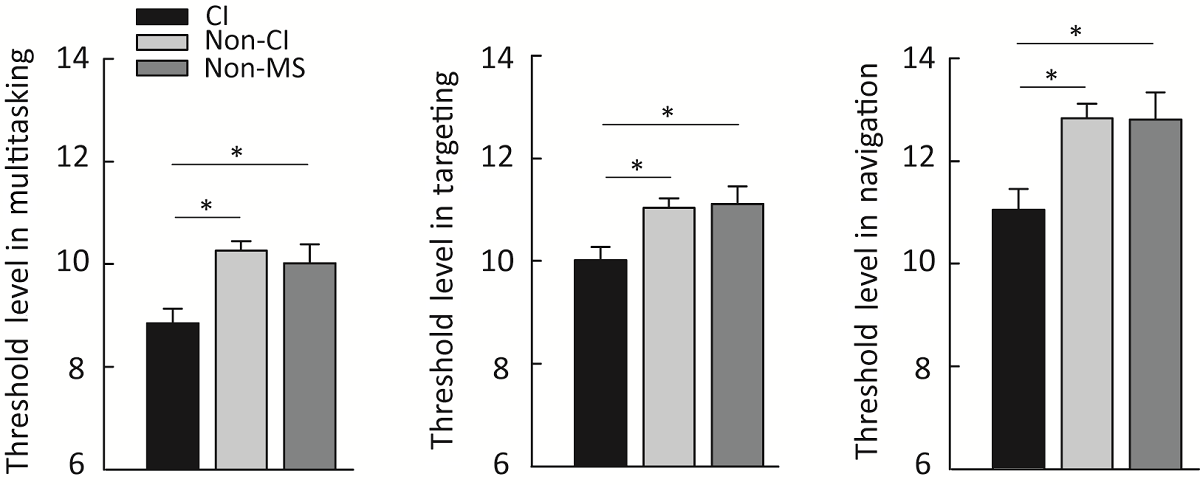
Group differences in EVO Monitor performance between CI, non-CI participants with MS and non-MS participants. Error bars represent standard error of the mean. CI: cognitive impairment; MS: multiple sclerosis. *P≤.01.

To evaluate whether EVO Monitor is sensitive to differences between participants with MS with and without CI as well as participants without MS, one-way ANCOVA with age, sex, years of education, and BRT as covariates was performed for the threshold level, which reflects task performance. Significant group differences in multitasking (F_2,109_=8.33, *P*<.001), perceptual discrimination (F_2,109_=5.63, *P*=.005), and visuomotor tracking (F_2,109_=6.97, *P*=.001) threshold level were found. Post hoc analyses showed a lower threshold level in participants with CI compared with both participants without CI with MS and participants without MS in all 3 conditions (multitasking: CI vs non-CI, 8.85 [SEM 0.28] vs 10.26 [SEM 0.19]; *P<.*001; CI vs non-MS, 8.85 [SEM 0.28] vs 10.01 [SEM 0.37]; *P=.*01; perceptual discrimination: CI vs non-CI, 10.01 [SEM 0.26] vs 11.04 [SEM 0.18]; *P*=.002; CI vs non-MS, 10.01 [SEM 0.26] vs 11.11 [SEM 0.34]; *P*=.01; visuomotor tracking: CI vs non-CI, 11.05 [SEM 0.40] vs 12.83 [SEM 0.28]; *P<.*001; CI vs non-MS, 11.05 [SEM 0.40] vs 12.81 [SEM 0.52]; *P*=.01) (Figure 3). These findings indicate that EVO Monitor, a video game–based assessment designed to assess executive function, attention, and information processing speed, is sensitive to capture group-level differences between participants with MS with or without CI as well as participants without MS.

### Association Between Performance in EVO Monitor and Standard Measures

**Figure 4:**
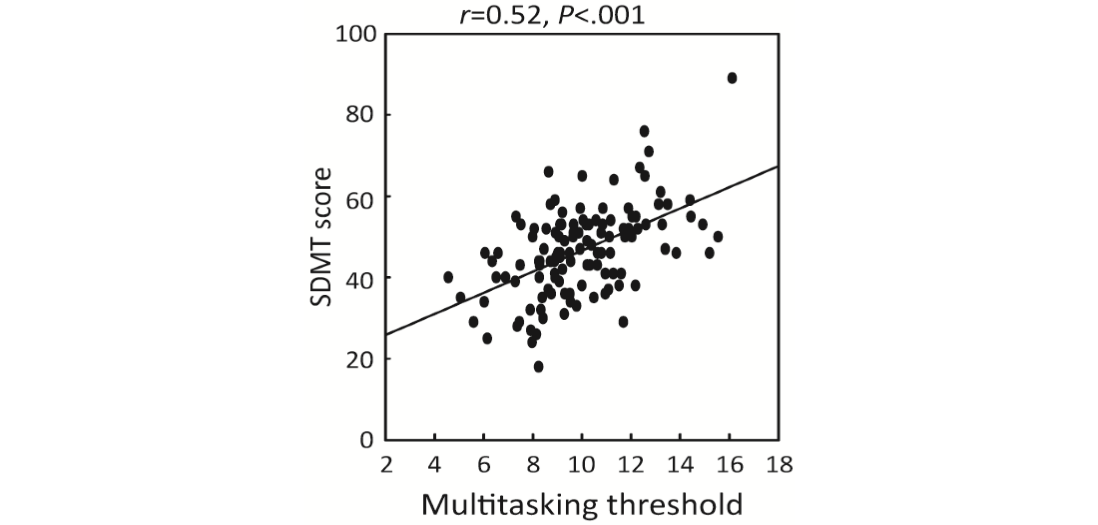
Correlation between EVO Monitor performance and SDMT score. SDMT: Symbol Digit Modalities Test.

Pearson correlation analyses were performed to scrutinize associations between performance in EVO Monitor and standard cognitive measures. The SDMT showed significant correlations with the EVO multitasking threshold level (Figure 4 and Table 2). Including age, sex, years of education, and BRT as covariates did not change the results (Table 2). Restricting the analyses to only participants with MS showed similar results. Furthermore, associations between clinical characteristics (ie, Expanded Disability Status Scale [EDSS] and disease duration) and the EVO multitasking threshold level were observed. Analyses of EVO perceptual discrimination and visuomotor tracking threshold levels showed similar results (Table 2).

**Table 2:** Correlation between performance in standard cognitive measures and EVO Monitor.

EVO measures, population, and standard measures				Covariates		*r*		*P* value
**Multitasking threshold level**								
	**All participants**							
		SDMT^a^	N/A^b^		0.52		<.001	
		SDMT	Age, sex, education (years), and BRT^c^		0.38		<.001	
	**Participants with MS^d^**							
		SDMT	N/A		0.46		<.001	
		SDMT	Age, sex, education (years), and BRT		0.41		<.001	
		EDSS^e^	N/A		–0.31		.002	
		Disease duration	N/A		–0.26		.01	
**Perceptual discrimination threshold level**								
	**All participants**							
		SDMT	N/A		0.50		<.001	
		SDMT	Age, sex, education (years), and BRT		0.40		<.001	
	**Participants with MS**							
		SDMT	N/A		0.39		<.001	
		SDMT	Age, sex, education (years), and BRT		0.34		.001	
		EDSS	N/A		–0.31		<.001	
		Disease duration	N/A		–0.29		.003	
**Visuomotor tracking** **threshold level**								
	**All participants**							
		SDMT	N/A		0.49		<.001	
		SDMT	Age, sex, education (years), and BRT		0.38		<.001	
	**Participants with MS**							
		SDMT	N/A		0.44		.01	
		SDMT	Age, sex, education (years), and BRT		0.40		<.001	
		EDSS	N/A		–0.25		.01	
		Disease duration	N/A		–0.21		.03	

^a^SDMT: Symbol Digit Modalities Test.

^b^N/A: not applicable.

^c^BRT: basic reaction time.

^d^MS: multiple sclerosis.

^e^EDSS: Expanded Disability Status Scale.

**Figure 5:**
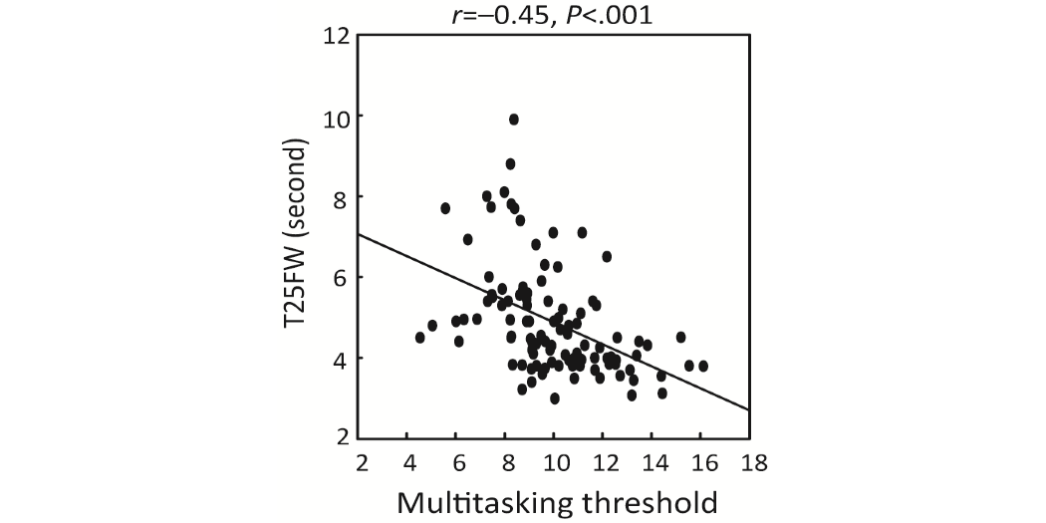
Correlation between EVO Monitor performance and T25FW. T25FW: Timed 25-Foot Walk.

**Figure 6:**
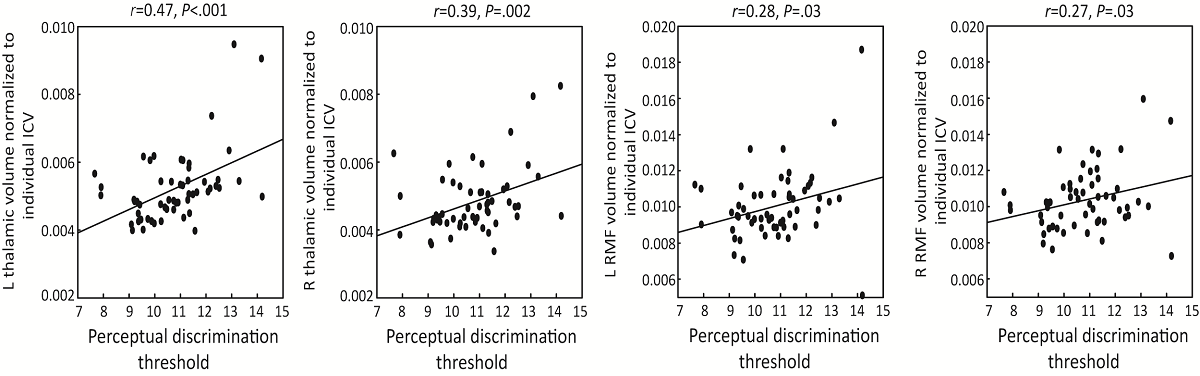
Correlation between EVO Monitor performance and magnetic resonance imaging volumetric data. ICV: intracranial volume; L: left; R: right; RMF: rostral middle frontal.

In a planned exploratory analysis, we investigated the correlation between performance in EVO Monitor and both MS-related physical function (walking speed, T25FW) and MRI volumetric data, given that these factors have been reported to associate with cognition in MS [31-34]. A negative correlation was observed between T25FW and multitasking threshold level (*r*=–0.45, *P*<.001) (Figure 5). The results remained similar when including demographic features as covariates (Table 3). Clinically acquired MRI volumetric data were available in 56 participants with MS (17 with CI). We focused on the thalamus and frontal lobes, given their reported relationships with cognition in MS [33,34]. The analyses revealed a positive correlation between the EVO perceptual discrimination threshold level and bilateral thalamic (left: *r*=0.47, *P*<.001; right: *r*=0.39, *P*=.002) as well as rostral middle frontal (left: *r*=0.28, *P*=.03; right: *r*=0.27, *P*=.03) volumes (Figure 6). The association with thalamic volumes persisted after adjusting for age, sex, years of education, and BRT (left: *r*=0.49, *P*<.001; right: *r*=0.38, *P*=.007). These results suggest that EVO Monitor performance is associated with standard MS cognitive and physical measures as well as MRI volumetric data.

**Table 3:** Results of correlation between physical measure (Timed 25-Foot Walk) and EVO Monitor performance.

EVO measures, population, and covariates			*r*		*P* value	
**Multitasking threshold level**						
	**All participants**					
		N/A^a^		–0.45		<.001
		Age, sex, education (years), and BRT^b^		–0.30		.003
	**Participants with MS^c^**					
		N/A		–0.41		<.001
		Age, sex, education (years), and BRT		–0.34		.002
**Perceptual discrimination threshold level**						
	**All participants**					
		N/A		–0.37		<.001
		Age, sex, education (years), and BRT		–0.22		.02
	**Participants with MS**					
		N/A		–0.29		.006
		Age, sex, education (years), and BRT		–0.21		.06
**Visuomotor tracking threshold level**						
	**All participants**					
		N/A		–0.48		<.001
		Age, sex, education (years), and BRT		–0.38		<.001
	**Participants with MS**					
		N/A		–0.43		<.001
		Age, sex, education (years), and BRT		–0.40		<.001

^a^N/A: not applicable.

^b^BRT: basic reaction time.

^c^MS: multiple sclerosis.

## Discussion

In this study, we aimed to determine whether in-game assessment features of EVO Monitor, an unsupervised, digital, video game–based tool integrated with adaptive algorithms, could represent a novel and sensitive way to perform unsupervised cognitive evaluations in MS. We found a significant group-level difference in performance on EVO Monitor among participants with MS with and without CI as well as participants without MS. Furthermore, we discovered an association between performance in EVO Monitor and standard cognitive measures, physical measures, and MRI volumetric data. These results provide evidence that EVO Monitor, an unsupervised, tablet game–based digital tool designed to assess executive function, attention, and information processing, could effectively assess cognitive performance in people with MS.

We demonstrated that EVO Monitor is sensitive enough to reveal group-level differences, as shown by a lower threshold level in participants with MS with CI compared to both participants with MS without CI and participants without MS in all 3 task conditions. The EVO Monitor program was developed to challenge nearly all aspects of cognition, including executive function, attention, and information processing. In perceptual discrimination and visuomotor tracking tasks, fast information processing is highly demanded, given that the participants are asked to make a fast response to certain stimuli (ie, colored target) and rapidly changing environment (ie, dynamically moving road with walls and obstacles), respectively. To successfully perform the multitasking challenge, there are additional cognitive requirements for selective attention, sustained attention, task switching, and goal management. Since processing speed, attention control, and executive function are the most commonly affected cognitive domains in MS [4,5], the lower EVO Monitor performance in participants with MS and CI may reflect a lower performance in these cognitive domains in general. These results suggest that cognitive dysfunction in MS can be captured by EVO Monitor, a digital tool with adaptive algorithms developed for cognitive assessment.

Using unsupervised digital tools provides a less stressful context and a high level of standardization of assessment. Moreover, it can be easily applied in multiple settings, including patients’ homes, which substantially improves access to these therapies for patients who may face scheduling, geographic, or economic barriers in accessing standard forms of cognitive assessment. With the advances in digital therapeutics, assessment and health care services for people with MS have been transitioning to digital platforms [25,26,52]. These results extended the application of digital tools for cognitive assessments in MS by incorporating advanced visualization and reward loops with closed-loop adaptation mechanics to enhance engagement and reduce interindividual variability, which leads to more reliable assessments [8,47].

An association between performance in EVO Monitor and standard measures was observed. Participants with a better performance in EVO Monitor showed higher SDMT scores. SDMT is considered the most sensitive measurement for the evaluation of cognitive involvement and information processing speed in the MS course [29,30]. While the participant substitutes geometric symbols for numbers while scanning a response key, a number of domains—processing speed, sustained attention, visual scanning and tracking, and motoric quickness—are being challenged. Although the task structures are different between EVO Monitor and SDMT, the shared cognitive functions subserving the two tasks may explain the correlational links. The results suggest that EVO Monitor can reliably reflect performance in cognitive tasks that involve information processing speed, selective attention, sustained attention, visual tracking, and goal management. Importantly, analyses including only participants with MS did not change the results but further revealed a negative correlation between clinical characteristics (ie, EDSS and disease duration) and performance in EVO Monitor, where participants with MS who had a longer disease duration and higher level of disability showed a lower performance in EVO Monitor. Although the correlation was modest, given that cognitive deficits tend to progress with disease duration [53] and disability progression [54] in MS, the observed association was expected.

In an exploratory analysis, we discovered an association between EVO Monitor performance and the T25FW as well as the bilateral rostral middle frontal and thalamic volume. In line with previous studies reporting that cognition in MS is associated with physical fitness and balance [55,56] and the concept of cognitive-motor coupling [57], in this study we found that better EVO Monitor performance is correlated with a faster walking speed as measured by the T25FW. Imaging [58] and postmortem [59] studies have suggested that frontal and subcortical regions involved in executive function and cognitive processing speed are also related to the spatial and temporal aspects of gait. The observed association between EVO Monitor performance and the T25FW may be explained by cerebral injury causing impairment in both domains in MS.

Additionally, the performance in the perceptual discrimination task of EVO Monitor positively correlated with rostral middle frontal and thalamic volume. The rostral middle frontal region is part of the dorsolateral prefrontal cortex, an area that is considered the center of executive function [60] and several domains of cognitive control abilities required to perform the EVO perceptual discrimination task, including sustained attention [61], selective attention [62], and inhibitory control [63]. Studies have also demonstrated that the thalamus mediates arousal states [64], and thalamic atrophy is the most significant MRI correlate of CI in MS [65,66]. It is unclear why performance in multitasking, a task including both perceptual discrimination and visuomotor tracking, did not correlate with thalamus and frontal volumetric data. Given that the posterior cortex has been linked to sustaining attention to spatial locations [67], a key component of the visuomotor task, it is possible that the multitasking task does not solely rely on frontal and thalamic resources but is also supported by the posterior cortex. Therefore, the examined correlation was not strong enough to be detected, as it was in the perceptual discrimination task.

Although our analyses were exploratory, the observed correlations support the need to better understand how EVO Monitor performance is associated with physical performance and the structural and functional changes of the brain in people with MS. Specifically, studies with functional MRI and connectivity data would provide essential information about neural correlates of the EVO Monitor tasks and, more importantly, pathological changes related to CI and neural plastic changes as a result of cognitive remediation.

Previously, we showed that EVO-AKL-T01, a video game–based digital tool similar to EVO Monitor, is an effective in-home cognitive remediation program for MS [17,18]. The high adherence rate during the 4- to 6-week home-based cognitive rehabilitation strategy indicated that remote digital tools are well accepted by patients with MS, who may have limited access to cognitive assessment or treatment. Since EVO Monitor is a digital tool designed as a self-guided assessment, it can be used as an in-home cognitive evaluation with multiple assessments to track either the progression of CI or the responsiveness to cognitive interventions, which can substantially help patients navigate problems related to cognitive issues. Future studies are warranted to evaluate the use of EVO Monitor in everyday situations across different contexts and investigate whether the results would be different due to fluctuations in cognitive ability during the day. Another key factor in cognitive assessment is patient fatigue. Well-designed studies that control for impacts of perceived fatigue and fatigability on cognitive assessments measured by digital tools are needed. Future studies comparing how different aspects of fatigue (eg, physical vs cognitive fatigue) affect cognitive performance as assessed by a comprehensive neuropsychological examination and by a digital tool that provides a shorter assessment time would provide additional insight into the role of fatigue in digital cognitive assessments.

There are some limitations to this study. Since we only included one time point in this cross-sectional analysis, it is difficult to determine the reproducibility of the observed results to conclude the test-retest reliability of EVO Monitor. In the exploratory analysis of the association between performance in EVO Monitor and MRI volumetric measures, clinically acquired MRI scans were only available for about half of the participants with MS (n=56), and the impact of acquisition protocol heterogeneity on our MRI metrics should be taken into account while a robust image processing pipeline was applied. The age difference between the non-MS group and non-CI MS group is one of the caveats in this study. However, in our analyses, age was included as a covariate to control for the potential influence of age difference on the results. Skills using digital tools may be a confounding factor influencing the results, as participants with better digital tool skills or more experience using tablet devices may have performed better. Future studies investigating digital tools should control for participants’ experience and skills using digital devices.

This study extended the application of digital tools for cognitive assessments in MS by incorporating built-in adaptive staircase algorithms to enhance engagement and mitigate interindividual variability. Furthermore, the encouraging findings suggest that EVO Monitor, an unsupervised, tablet game–based program, is a clinically valuable approach to capturing CI in MS. Since CI is one of the most debilitating manifestations of MS and traveling to clinics may be burdensome due to deficits in mobility or cognition, some patients may have limited access to cognitive assessments. The application of digital cognitive assessments provides flexibility, as the testing can be performed in different settings, including patients’ homes. The development of digital cognitive assessments helps patients effectively detect CI and navigate cognition-related problems in their daily living. Future studies with multiple points of data collection and a deeper investigation of how physical performance and the functional and structural changes of the brain affect cognitive performance as measured by digital tools are warranted to provide additional insight.

## Abbreviations

ANCOVA: analysis of covariance
BRT: basic reaction time
CI: cognitive impairment
EDSS: Expanded Disability Status Scale
MRI: magnetic resonance imaging
MS: multiple sclerosis
SDMT: Symbol Digit Modalities Test
SEM: standard error of the mean
T25FW: Timed 25-Foot Walk
UCSF: University of California, San Francisco

## References

[1] Rao SM, Leo GJ, Ellington L, Nauertz T, Bernardin L, Unverzagt F (1991). Cognitive dysfunction in multiple sclerosis. II. Impact on employment and social functioning. Neurology.

[2] Ruet A, Deloire M, Hamel D, Ouallet J, Petry K, Brochet B (2013). Cognitive impairment, health-related quality of life and vocational status at early stages of multiple sclerosis: a 7-year longitudinal study. J Neurol.

[3] Amato MP, Langdon D, Montalban X, Benedict RHB, DeLuca J, Krupp LB, Thompson AJ, Comi G (2013). Treatment of cognitive impairment in multiple sclerosis: position paper. J Neurol.

[4] Achiron A, Barak Y (2003). Cognitive impairment in probable multiple sclerosis. J Neurol Neurosurg Psychiatry.

[5] Rogers JM, Panegyres PK (2007). Cognitive impairment in multiple sclerosis: evidence-based analysis and recommendations. J Clin Neurosci.

[6] Bobholz JA, Rao SM (2003). Cognitive dysfunction in multiple sclerosis: a review of recent developments. Curr Opin Neurol.

[7] Langdon DW (2011). Cognition in multiple sclerosis. Curr Opin Neurol.

[8] Anguera JA, Brandes-Aitken AN, Rolle CE, Skinner SN, Desai SS, Bower JD, Martucci WE, Chung WK, Sherr EH, Marco EJ (2016). Characterizing cognitive control abilities in children with 16p11.2 deletion using adaptive 'video game' technology: a pilot study. Transl Psychiatry.

[9] Anguera JA, Brandes-Aitken AN, Antovich AD, Rolle CE, Desai SS, Marco EJ (2017). A pilot study to determine the feasibility of enhancing cognitive abilities in children with sensory processing dysfunction. PLoS One.

[10] Anguera JA, Gunning FM, Areán Patricia A (2017). Improving late life depression and cognitive control through the use of therapeutic video game technology: A proof-of-concept randomized trial. Depress Anxiety.

[11] Anguera JA, Jordan JT, Castaneda D, Gazzaley A, Areán PA (2016). Conducting a fully mobile and randomised clinical trial for depression: access, engagement and expense. BMJ Innov.

[12] Arean PA, Hallgren KA, Jordan JT, Gazzaley A, Atkins DC, Heagerty PJ, Anguera JA (2016). The Use and Effectiveness of Mobile Apps for Depression: Results From a Fully Remote Clinical Trial. J Med Internet Res.

[13] Areàn PA, Hoa Ly K, Andersson G (2016). Mobile technology for mental health assessment. Dialogues Clin Neurosci.

[14] Charvet LE, Yang J, Shaw MT, Sherman K, Haider L, Xu J, Krupp LB (2017). Cognitive function in multiple sclerosis improves with telerehabilitation: Results from a randomized controlled trial. PLoS One.

[15] Davis NO, Bower J, Kollins SH (2018). Proof-of-concept study of an at-home, engaging, digital intervention for pediatric ADHD. PLoS One.

[16] Flynn RM, Colón-Acosta N, Zhou J, Bower J (2019). A Game-Based Repeated Assessment for Cognitive Monitoring: Initial Usability and Adherence Study in a Summer Camp Setting. J Autism Dev Disord.

[17] Bove R, Rowles W, Zhao C, Anderson A, Friedman S, Langdon D, Alexander A, Sacco S, Henry R, Gazzaley A, Feinstein A, Anguera JA (2020). A novel in-home digital treatment to improve processing speed in people with multiple sclerosis: A pilot study. Mult Scler.

[18] Bove RM, Rush G, Zhao C, Rowles W, Garcha P, Morrissey J, Schembri A, Alailima T, Langdon D, Possin K, Gazzaley A, Feinstein A, Anguera J (2019). A Videogame-Based Digital Therapeutic to Improve Processing Speed in People with Multiple Sclerosis: A Feasibility Study. Neurol Ther.

[19] Lee G (2013). Effects of training using video games on the muscle strength, muscle tone, and activities of daily living of chronic stroke patients. J Phys Ther Sci.

[20] Webster D, Celik O (2014). Systematic review of Kinect applications in elderly care and stroke rehabilitation. J Neuroeng Rehabil.

[21] Trapp W, Landgrebe M, Hoesl K, Lautenbacher S, Gallhofer B, Günther W, Hajak G (2013). Cognitive remediation improves cognition and good cognitive performance increases time to relapse – results of a 5 year catamnestic study in schizophrenia patients. BMC Psychiatry.

[22] Kollins SH, Bower J, Findling RL, Keefe R, Epstein J, Cutler AJ, White R, Aberle L, DeLoss D, Faraone SV (2018). 2.40 A Multicenter, Randomized, Active-Control Registration Trial of Software Treatment for Actively Reducing Severity of ADHD (Stars-Adhd) to Assess the Efficacy and Safety of a Novel, Home-Based, Digital Treatment for Pediatric ADHD. J Am Acad Child Adolesc Psychiatry.

[23] Kollins S, DeLoss D, Cañadas E, Lutz J, Findling R, Keefe R, Epstein JN, Cutler AJ, Faraone SV (2020). A novel digital intervention for actively reducing severity of paediatric ADHD (STARS-ADHD): a randomised controlled trial. Lancet Digital Health.

[24] Yerys BE, Bertollo JR, Kenworthy L, Dawson G, Marco EJ, Schultz RT, Sikich L (2019). Brief Report: Pilot Study of a Novel Interactive Digital Treatment to Improve Cognitive Control in Children with Autism Spectrum Disorder and Co-occurring ADHD Symptoms. J Autism Dev Disord.

[25] Finkelstein J, Lapshin O, Castro H, Cha E, Provance PG (2008). Home-based physical telerehabilitation in patients with multiple sclerosis: a pilot study. J Rehabil Res Dev.

[26] Hatzakis M, Haselkorn J, Williams R, Turner A, Nichol P (2003). Telemedicine and the delivery of health services to veterans with multiple sclerosis. J Rehabil Res Dev.

[27] Kane RL, Bever CT, Ehrmantraut M, Forte A, Culpepper WJ, Wallin MT (2008). Teleneurology in patients with multiple sclerosis: EDSS ratings derived remotely and from hands-on examination. J Telemed Telecare.

[28] Anguera JA, Boccanfuso J, Rintoul JL, Al-Hashimi O, Faraji F, Janowich J, Kong E, Larraburo Y, Rolle C, Johnston E, Gazzaley A (2013). Video game training enhances cognitive control in older adults. Nature.

[29] Benedict RH, DeLuca J, Phillips G, LaRocca N, Hudson LD, Rudick R, Multiple Sclerosis Outcome Assessments Consortium (2017). Validity of the Symbol Digit Modalities Test as a cognition performance outcome measure for multiple sclerosis. Mult Scler.

[30] Parmenter BA, Weinstock-Guttman B, Garg N, Munschauer F, Benedict RHB (2007). Screening for cognitive impairment in multiple sclerosis using the Symbol digit Modalities Test. Mult Scler.

[31] Sandroff BM, Hillman CH, Motl RW (2015). Aerobic fitness is associated with inhibitory control in persons with multiple sclerosis. Arch Clin Neuropsychol.

[32] Sandroff BM, Pilutti LA, Benedict RHB, Motl RW (2015). Association between physical fitness and cognitive function in multiple sclerosis: does disability status matter?. Neurorehabil Neural Repair.

[33] Rocca MA, Amato MP, De Stefano N, Enzinger C, Geurts JJ, Penner I, Rovira A, Sumowski JF, Valsasina P, Filippi M, MAGNIMS Study Group (2015). Clinical and imaging assessment of cognitive dysfunction in multiple sclerosis. Lancet Neurol.

[34] Rocca MA, Riccitelli GC, Meani A, Pagani E, Del Sette P, Martinelli V, Comi G, Falini A, Filippi M (2019). Cognitive reserve, cognition, and regional brain damage in MS: A 2 -year longitudinal study. Mult Scler.

[35] Polman CH, Reingold SC, Banwell B, Clanet M, Cohen JA, Filippi M, Fujihara K, Havrdova E, Hutchinson M, Kappos L, Lublin FD, Montalban X, O'Connor P, Sandberg-Wollheim M, Thompson AJ, Waubant E, Weinshenker B, Wolinsky JS (2011). Diagnostic criteria for multiple sclerosis: 2010 revisions to the McDonald criteria. Ann Neurol.

[36] Corfield F, Langdon D (2018). A Systematic Review and Meta-Analysis of the Brief Cognitive Assessment for Multiple Sclerosis (BICAMS). Neurol Ther.

[37] Langdon D, Amato M, Boringa J, Brochet B, Foley F, Fredrikson S, Hämäläinen P, Hartung H, Krupp L, Penner I, Reder A, Benedict R (2012). Recommendations for a Brief International Cognitive Assessment for Multiple Sclerosis (BICAMS). Mult Scler.

[38] Delis D, Kramer J, Kaplan E, Ober B (2000). California verbal learning test-II. 2nd ed.

[39] Benedict RH (1997). Brief visuospatial memory test - revised: professional manual.

[40] Benedict RHB, Duquin JA, Jurgensen S, Rudick RA, Feitcher J, Munschauer FE, Panzara MA, Weinstock-Guttman B (2008). Repeated assessment of neuropsychological deficits in multiple sclerosis using the Symbol Digit Modalities Test and the MS Neuropsychological Screening Questionnaire. Mult Scler.

[41] Cohen JA, Reingold SC, Polman CH, Wolinsky JS (2012). Disability outcome measures in multiple sclerosis clinical trials: current status and future prospects. The Lancet Neurology.

[42] Gronwall DM (1977). Paced auditory serial-addition task: a measure of recovery from concussion. Percept Mot Skills.

[43] García-Pérez MA (2014). Adaptive psychophysical methods for nonmonotonic psychometric functions. Atten Percept Psychophys.

[44] King-Smith PE, Rose D (1997). Principles of an adaptive method for measuring the slope of the psychometric function. Vision Res.

[45] Leek MR (2001). Adaptive procedures in psychophysical research. Percept Psychophys.

[46] Klein SA (2001). Measuring, estimating, and understanding the psychometric function: a commentary. Percept Psychophys.

[47] Mishra J, Anguera JA, Gazzaley A (2016). Video Games for Neuro-Cognitive Optimization. Neuron.

[48] Tustison NJ, Cook PA, Klein A, Song G, Das SR, Duda JT, Kandel BM, van Strien N, Stone JR, Gee JC, Avants BB (2014). Large-scale evaluation of ANTs and FreeSurfer cortical thickness measurements. Neuroimage.

[49] Klein A, Ghosh SS, Bao FS, Giard J, Häme Y, Stavsky E, Lee N, Rossa B, Reuter M, Chaibub Neto E, Keshavan A (2017). Mindboggling morphometry of human brains. PLoS Comput Biol.

[50] Whitwell JL, Crum WR, Watt HC, Fox NC (2001). Normalization of cerebral volumes by use of intracranial volume: implications for longitudinal quantitative MR imaging. AJNR Am J Neuroradiol.

[51] Kiely KM, Butterworth P, Watson N, Wooden M (2014). The Symbol Digit Modalities Test: Normative data from a large nationally representative sample of Australians. Arch Clin Neuropsychol.

[52] Hoang P, Schoene D, Gandevia S, Smith S, Lord SR (2016). Effects of a home-based step training programme on balance, stepping, cognition and functional performance in people with multiple sclerosis--a randomized controlled trial. Mult Scler.

[53] Amato MP, Ponziani G, Siracusa G, Sorbi S (2001). Cognitive dysfunction in early-onset multiple sclerosis: a reappraisal after 10 years. Arch Neurol.

[54] Carotenuto A, Moccia M, Costabile T, Signoriello E, Paolicelli D, Simone M, Lus G, Brescia Morra V, Lanzillo R, Cogniped study group (2019). Associations between cognitive impairment at onset and disability accrual in young people with multiple sclerosis. Sci Rep.

[55] Batista S, Teter B, Sequeira K, Josyula S, Hoogs M, Ramanathan M, Benedict RHB, Weinstock-Guttman B (2012). Cognitive impairment is associated with reduced bone mass in multiple sclerosis. Mult Scler.

[56] Sandroff BM, Motl RW (2012). Fitness and cognitive processing speed in persons with multiple sclerosis: a cross-sectional investigation. J Clin Exp Neuropsychol.

[57] Benedict RHB, Holtzer R, Motl RW, Foley FW, Kaur S, Hojnacki D, Weinstock-Guttman B (2011). Upper and lower extremity motor function and cognitive impairment in multiple sclerosis. J Int Neuropsychol Soc.

[58] Rosano C, Aizenstein HJ, Wu M, Newman AB, Becker JT, Lopez OL, Kuller LH (2007). Focal atrophy and cerebrovascular disease increase dementia risk among cognitively normal older adults. J Neuroimaging.

[59] Whitman GT, Tang Y, Lin A, Baloh RW, Tang T (2001). A prospective study of cerebral white matter abnormalities in older people with gait dysfunction. Neurology.

[60] Alvarez JA, Emory E (2006). Executive function and the frontal lobes: a meta-analytic review. Neuropsychol Rev.

[61] Ortuño F, Ojeda N, Arbizu J, López P, Martí-Climent JM, Peñuelas I, Cervera S (2002). Sustained attention in a counting task: normal performance and functional neuroanatomy. Neuroimage.

[62] Hadland KA, Rushworth MF, Passingham RE, Jahanshahi M, Rothwell JC (2001). Interference with performance of a response selection task that has no working memory component: an rTMS comparison of the dorsolateral prefrontal and medial frontal cortex. J Cogn Neurosci.

[63] Hoppenbrouwers SS, De Jesus DR, Stirpe T, Fitzgerald PB, Voineskos AN, Schutter DJLG, Daskalakis ZJ (2013). Inhibitory deficits in the dorsolateral prefrontal cortex in psychopathic offenders. Cortex.

[64] Van der Werf YD, Witter MP, Groenewegen HJ (2002). The intralaminar and midline nuclei of the thalamus. Anatomical and functional evidence for participation in processes of arousal and awareness. Brain Res Brain Res Rev.

[65] Batista S, Zivadinov R, Hoogs M, Bergsland N, Heininen-Brown M, Dwyer MG, Weinstock-Guttman B, Benedict RHB (2012). Basal ganglia, thalamus and neocortical atrophy predicting slowed cognitive processing in multiple sclerosis. J Neurol.

[66] Benedict RHB, Bruce JM, Dwyer MG, Abdelrahman N, Hussein S, Weinstock-Guttman B, Garg N, Munschauer F, Zivadinov R (2006). Neocortical atrophy, third ventricular width, and cognitive dysfunction in multiple sclerosis. Arch Neurol.

[67] Malhotra P, Coulthard EJ, Husain M (2009). Role of right posterior parietal cortex in maintaining attention to spatial locations over time. Brain.

