# Correction: Application of an Adaptive, Digital, Game-Based Approach for Cognitive Assessment in Multiple Sclerosis: Observational Study

**DOI:** 10.2196/27440

**Published:** 2021-01-27

**Authors:** Wan-Yu Hsu, William Rowles, Joaquin A Anguera, Chao Zhao, Annika Anderson, Amber Alexander, Simone Sacco, Roland Henry, Adam Gazzaley, Riley Bove

**Affiliations:** 1 Department of Neurology Weill Institute for Neurosciences University of California, San Francisco San Francisco, CA United States; 2 Neuroscape University of California, San Francisco San Francisco, CA United States; 3 Department of Psychiatry University of California, San Francisco San Francisco, CA United States; 4 Department of Physiology University of California, San Francisco San Francisco, CA United States

In “Application of an Adaptive, Digital, Game-Based Approach for Cognitive Assessment in Multiple Sclerosis: Observational Study” (J Med Internet Res 2021;23(1):e24356) the authors noted two errors.

In the originally published manuscript, author *Joaquin Anguera* was incorrectly named. The following name has been included in the corrected version of the manuscript: *Joaquin A Anguera*.

In the originally published manuscript, a copyright credit was not included in the caption of Figure 1. The following text has been added to the end of the Figure 1 caption in the corrected version of the manuscript:

Copyright © 2020-2021, Akili Interactive Labs, Inc. All rights reserved.

The corrections will appear in the online version of the paper on the JMIR Publications website on January 27, 2021, together with the publication of this correction notice. Because this was made after submission to PubMed, PubMed Central, and other full-text repositories, the corrected article has also been resubmitted to those repositories.

